# Supplementary figures and images for: The farther, the better? The effect of attentional focus distance on motor performance: a systematic review and meta-analysis
Source: PeerJ. 2025 Sep 8;13:e20012. doi: 10.7717/peerj.20012 (PMC12424610; doi:10.7717/peerj.20012)

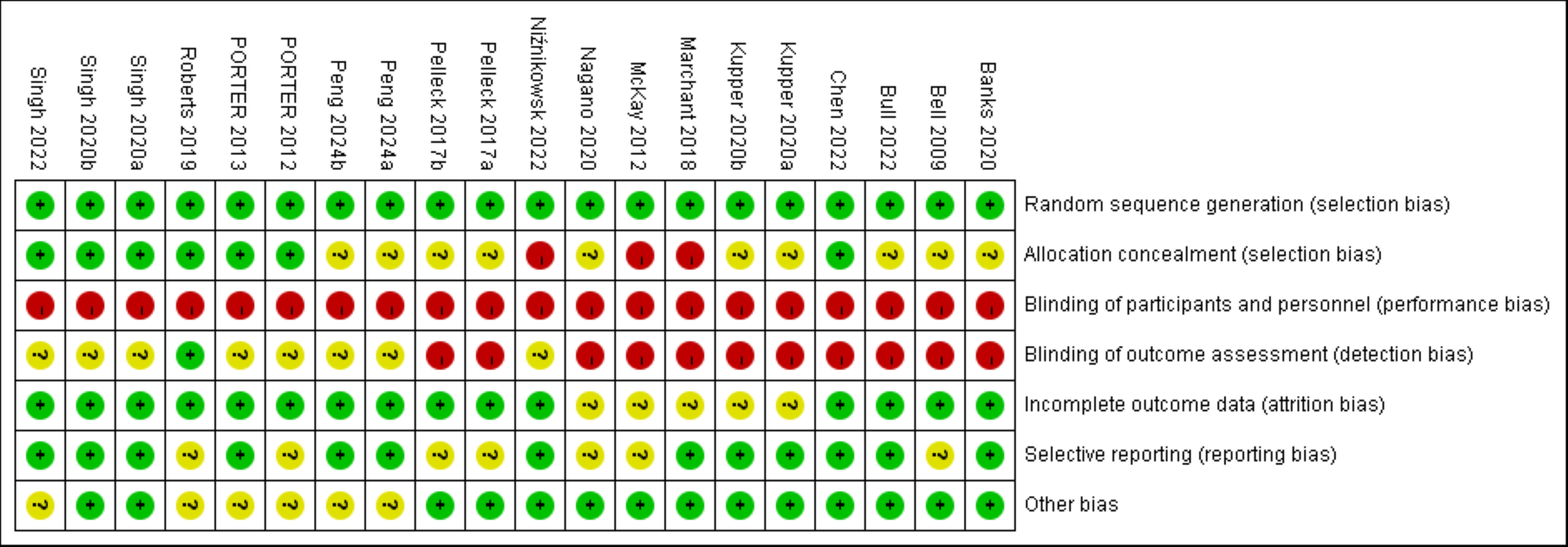

Supplement: Supplemental Information 2 [file peerj-13-20012-s002.png]

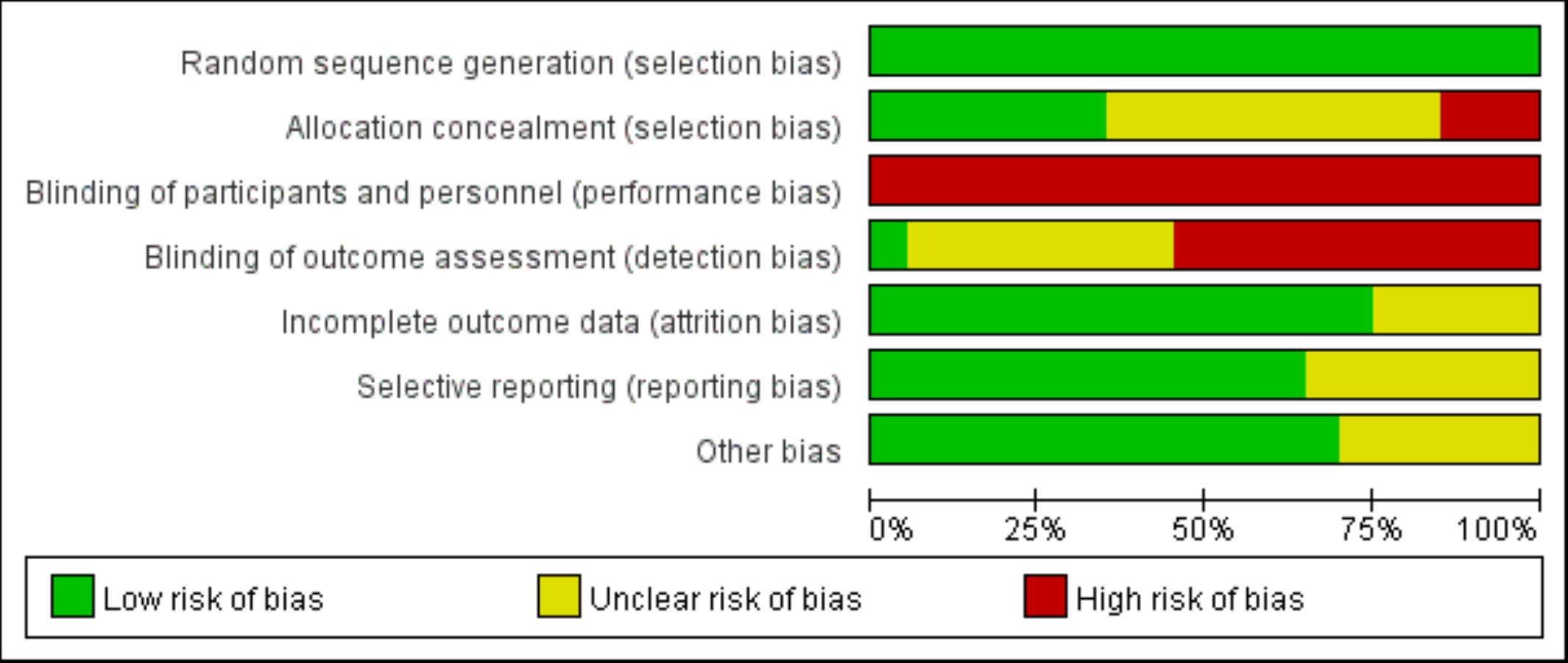

Supplement: Supplemental Information 3 [file peerj-13-20012-s003.png]

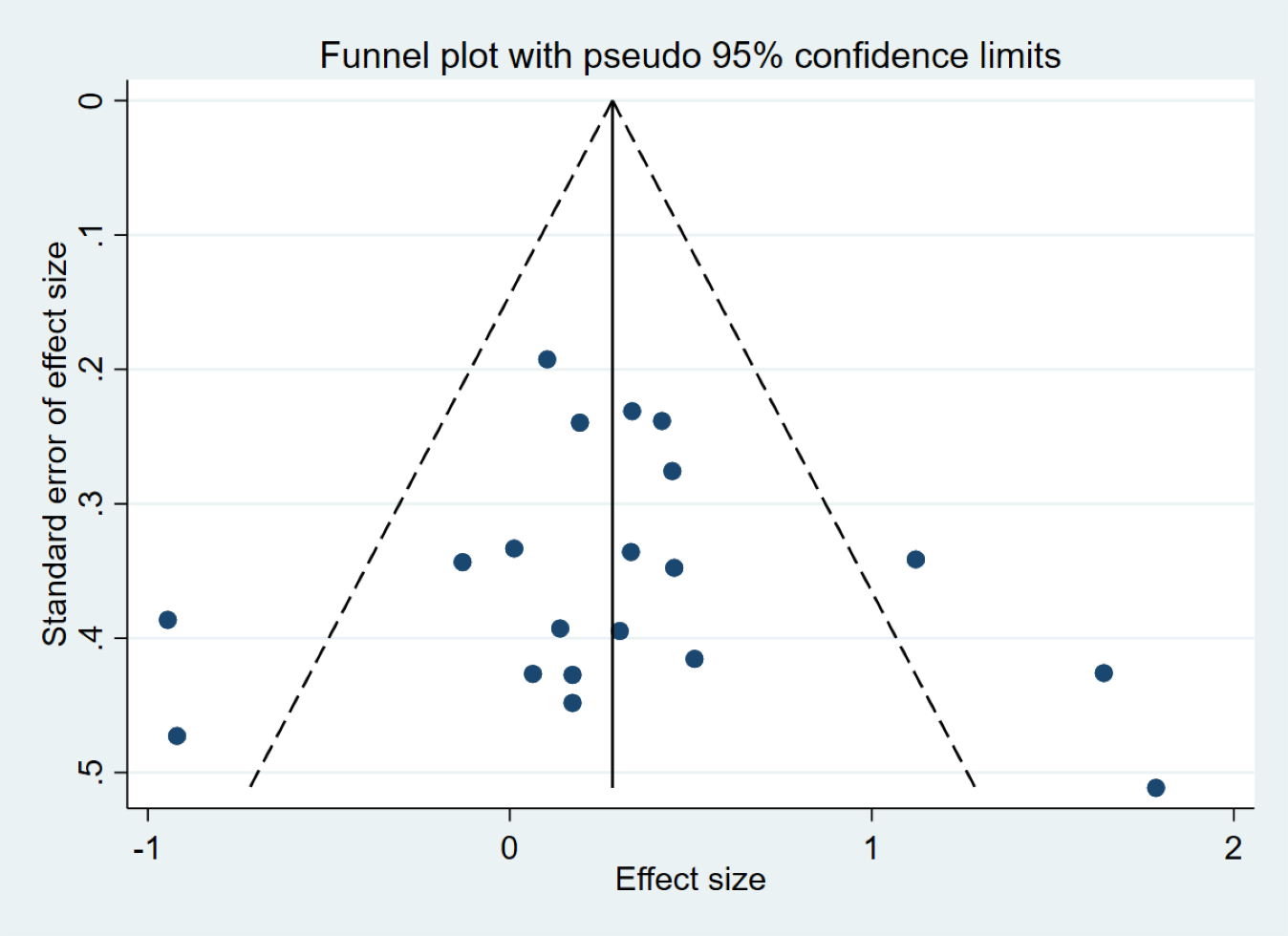

Supplement: Supplemental Information 4 [file peerj-13-20012-s004.png]

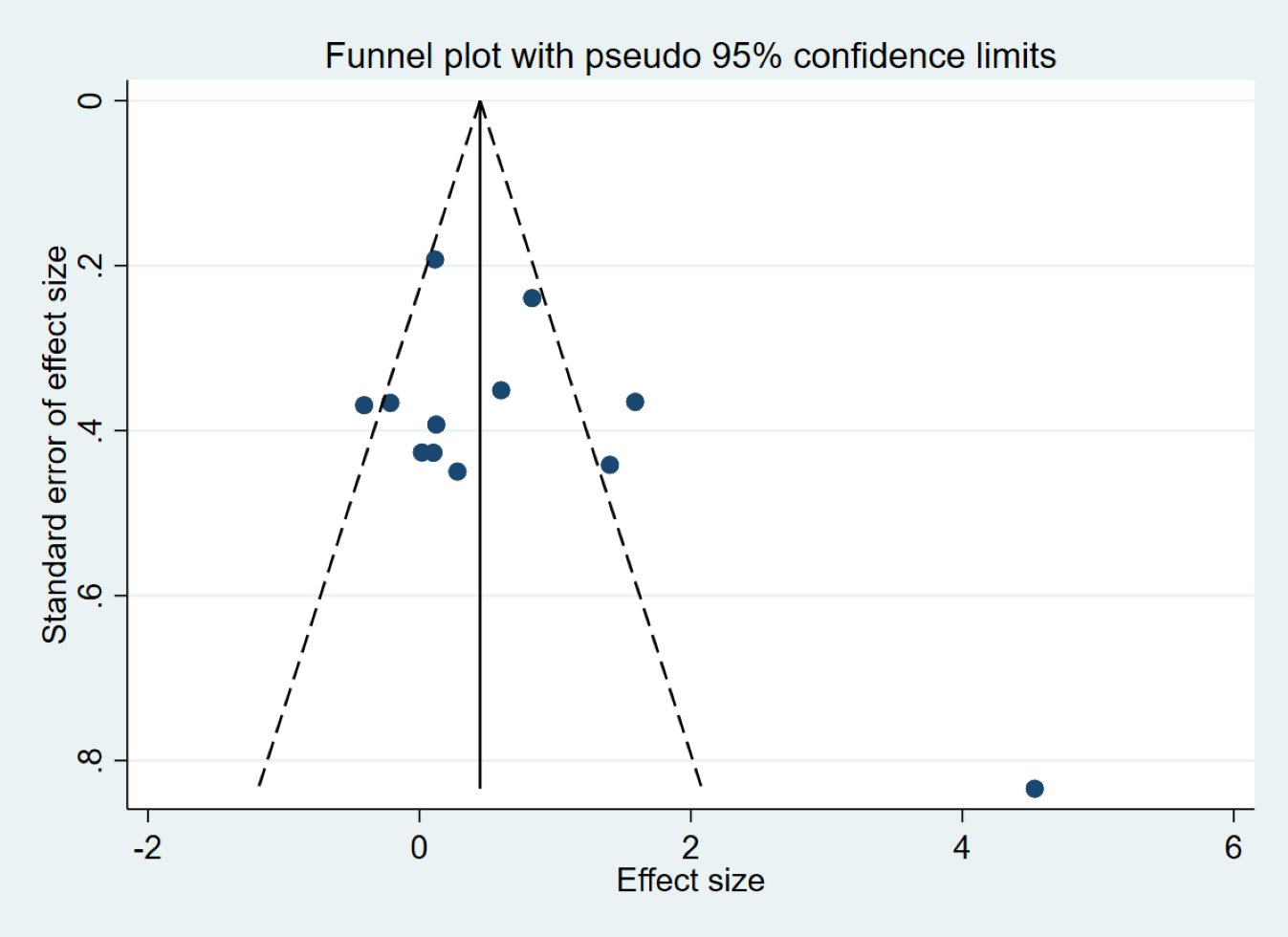

Supplement: Supplemental Information 5 [file peerj-13-20012-s005.png]

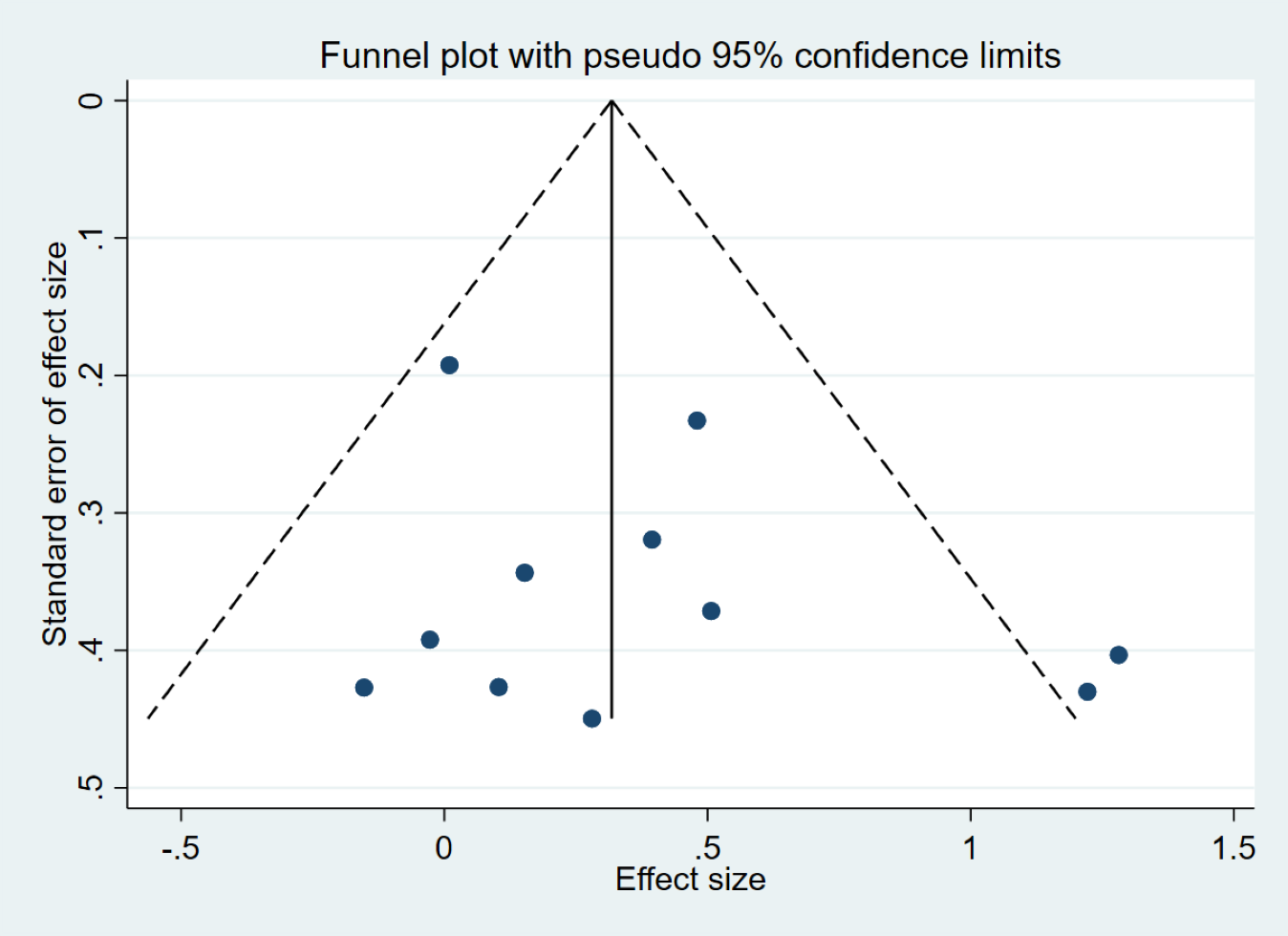

Supplement: Supplemental Information 6 [file peerj-13-20012-s006.png]
